# A pilot study suggests the correspondence between SAR202 bacteria and dissolved organic matter in the late stage of a year-long microcosm incubation

**DOI:** 10.3389/fmicb.2024.1357822

**Published:** 2024-04-03

**Authors:** Yufeng Jia, Changfei He, Madeline Lahm, Qi Chen, Leanne Powers, Michael Gonsior, Feng Chen

**Affiliations:** ^1^Institute of Marine and Environmental Technology, University of Maryland Center for Environmental Science, Baltimore, MD, United States; ^2^State Key Laboratory for Marine Environmental Science, Institute of Marine Microbes and Ecospheres, College of Ocean and Earth Sciences, Xiamen University, Xiamen, China; ^3^Chesapeake Biological Laboratory, University of Maryland Center for Environmental Science, Solomons, MD, United States; ^4^State University of New York College of Environmental Science and Forestry, Department of Chemistry, Syracuse, NY, United States

**Keywords:** SAR202, recalcitrant DOM, niche specification, network analysis, long-term incubation

## Abstract

SAR202 bacteria are abundant in the marine environment and they have been suggested to contribute to the utilization of recalcitrant organic matter (RDOM) within the ocean’s biogeochemical cycle. However, this functional role has only been postulated by metagenomic studies. During a one-year microcosm incubation of an open ocean microbial community with lysed *Synechococcus* and its released DOM, SAR202 became relatively more abundant in the later stage (after day 30) of the incubation. Network analysis illustrated a high degree of negative associations between SAR202 and a unique group of molecular formulae (MFs) in phase 2 (day 30 to 364) of the incubation, which is empirical evidence that SAR202 bacteria are major consumers of the more oxygenated, unsaturated, and higher-molecular-weight MFs. Further investigation of the SAR202-associated MFs suggested that they were potentially secondary products arising from initial heterotrophic activities following the amendment of labile *Synechococcus*-derived DOM. This pilot study provided a preliminary observation on the correspondence between SAR202 bacteria and more resistant DOM, further supporting the hypothesis that SAR202 bacteria play important roles in the degradation of RDOM and thus the ocean’s biogeochemical cycle.

## Introduction

Marine microorganisms contribute significantly to ocean carbon and nutrient cycling (Azam et al., 1983; Jiao et al., 2010, 2018; Moran et al., 2016; Zhang et al., 2018). A significant amount of organic carbon fixed by phytoplankton is utilized by microorganisms and transformed into more resistant dissolved organic matter (DOM) in the deep ocean through the process of the microbial carbon pump (Jiao et al., 2010). Oceans store a large pool of reduced dissolved organic carbon which is nearly equivalent to the carbon inventory (carbon dioxide) in the atmosphere (Hansell et al., 2009). DOM in seawater is complex and contains millions of different chemical molecules (Dittmar, 2015). To better understand the utilization of DOM by microorganisms, DOM has been operationally divided into labile, semi-labile, and refractory/recalcitrant pools based on their bioavailability (Kirchman et al., 1993). The incubation of phytoplankton DOM with the natural microbial community has been a common way to estimate the turnover time of these three DOM pools by microorganisms. In general, the labile DOM is consumed by microbes in the first few days, semi-labile in a few weeks, and recalcitrant in months and years (Hansell, 2013).

The SAR202 bacteria were originally discovered at the Bermuda Atlantic Time-series (BATS) station (Giovannoni et al., 1996). They are abundant below the deep chlorophyll maximum (DeLong et al., 2006), and become more abundant in the deeper ocean (Schattenhofer et al., 2009). SAR202 bacteria can contribute up to 30% of the microbial community in the mesopelagic and bathypelagic waters (Morris et al., 2004; Varela et al., 2008). The single-amplified genomes of SAR202 indicated that the oxidation of resistant molecules that accumulate in the marine DOM pool is feasible due to the presence of high numbers of flavin mononucleotide monooxygenases (FMNOs), various short-chain dehydrogenases, and other enzymes in SAR202 (Landry et al., 2017). These oxidative enzymes encoded in SAR202 have been proposed to contribute to the transformation of less labile molecules to more recalcitrant DOM in the ocean (Landry et al., 2017). Metatranscriptomic data confirmed SAR202’s ability to degrade recalcitrant compounds, shown by active oxidative enzymes such as alkanal monooxygenase and catechol 2,3-dioxygenase (Gao et al., 2019). Multiple metagenomic and metatranscriptomic data of SAR202 from the deep ocean have suggested that SAR202 bacteria are versatile for degrading semi-labile or recalcitrant DOM in the ocean (Landry et al., 2017; Colatriano et al., 2018; Gao et al., 2019; Saw et al., 2020; Wei et al., 2020). Moreover, SAR202 bacteria have the capability to assimilate ammonia, degrade osmolytes and organosulfur, and synthesize VB_12_ (Mehrshad et al., 2018; Wei et al., 2020). Despite multiple reports of predicted SAR202’s metabolic pathways, no empirical data to date is available to connect SAR202 with the degradation of recalcitrant DOM.

The laboratory incubation approach has been widely used to investigate the response of pure cultures or microbial communities to certain substrates. The *in vitro* observation is more controllable over an extensive period and easier to collect samples than the *in situ* study. Recently, DOM derived from picocyanobacteria has been added to marine microbial community to investigate the response of the microorganisms and their role in the utilization and transformation of cyanobacterial DOM (Zhao et al., 2019; Zheng et al., 2019; Xie et al., 2020; Zheng et al., 2021; Wang et al., 2022). Xie et al. (2020) reported an increased abundance of Chloroflexi (SAR202) in the later stage of incubation and they divided the incubation period (total 180 days) into three phases corresponding to labile, semi-lable, and recalcitrant stage of DOM. However, no correlations between SAR202 bacteria and DOM molecules were investigated in this study (Xie et al., 2020).

Correlation-based network analysis is valuable for data mining and visualization *in silico* analysis of large datasets (Batushansky et al., 2016), and it can provide insights into the dynamics of microbial community structures (Cardona et al., 2016). Several incubation studies have used network analysis to illustrate complex relationship between microbial taxa and DOM molecules based on 16S rRNA gene sequences and ultra-high resolution mass spectrometry data (Luria et al., 2017; Zhou et al., 2018; Zhao et al., 2019; Xiao et al., 2021; Zhou et al., 2021; Raina et al., 2022; Wang et al., 2022). Based on the same incubation experiment (coastal water) used by Xie et al. (2020), Wang et al. (2022) reported that the OTUs from the SAR202 clade had a relatively high degree of associations with DOM in the later stage of incubation, yet no detailed relationships between individual SAR202 groups and molecular formulae were explored and the role of SAR202 was only briefly mentioned. Furthermore, molecular properties of SAR202-associated DOM have not been explored.

Here, we applied in-depth statistical analysis to explore the relationship between SAR202 and the chemical heterogeneity of DOM in a microcosm experiment. The *Synechococcus* DOM was added to the microbial community collected from the oligotrophic ocean. High-degree correlations between SAR202 bacteria and DOM molecular formulae (MFs) were observed after 30 days incubation. By examining the associations for SAR202 bacteria and their correlated MFs, we provide substantial organic chemical evidence to the hypothesis stemming from recent metagenomic studies that SAR202 bacteria have the specialized ability to degrade complex and recalcitrant DOM in the ocean.

## Materials and methods

### Incubation setup and subsampling

The incubation setup and subsampling procedures were previously reported in the supplemental information of Jia et al. (2023). Briefly, seawater was collected from the North Atlantic Gulf Stream (34° 9′ 33.73″ N, 77° 43′ 57.73″ W, surface water) and filtered through GF/F filters (Whatman®) to obtain the microbial community for incubations. The treatment triplicates were amended with pre-filtered *Synechococcus*-derived DOM (Syn-DOM) on day 0 and no alteration was applied to the control triplicates. All incubations were kept in the dark with ventilation at room temperature for 1 year. Subsamples were taken on day 0, 1, 3, 10, 30, 60, 90, and 364 for analyses of microbial abundance, microbial community composition, and organic compound composition. Additional subsamples on day 5, 7, 15, 22, 75, and 180 were taken for analyses of microbial abundance and community composition.

### High-throughput 16S rRNA sequencing

Microbial DNA was extracted from 0.2 μm filters of each subsample, using the phenol/chloroform protocol described elsewhere (Kan, 2006). Thawed filter was placed in a Whirl-Pak bag, and 2 mL lysis buffer (0.1 M Tris–HCl, pH = 8.0; 0.1 M EDTA; 0.8 M sucrose) and 10 μL lysozyme (200 μg/μL) were added into the bag. The Whirl-Pak bag was incubated at 37°C for 30 min, amended with 10 μL proteinase K (20 mg/mL) and 10 μL SDS (final concentration 1%), and further incubated at 37°C overnight (Kan et al., 2006). The solution was added 100 μL CTAB + NaCl (10%, 1.4 M) and incubated at 65°C for 30 min. DNA was partitioned with phenol:chloroform:isoamyl alcohol (25:24:1, v/v) and chloroform:isoamyl alcohol (24,1, v/v) and precipitated by isopropanol. DNA precipitate was washed with cold ethanol (4°C, 70%) and eluted with nuclease-free water.

The V4 region of microbial 16S rRNA gene was amplified with PCR using forward primer 515F (5′-GTGYCAGCMGCCGCGGTAA-3′; Parada et al., 2016) and reverse primer 806R (5′-GGACTACNVGGGTWTCTAAT-3′; Apprill et al., 2015). High-throughput DNA sequencing was performed on the Illumina MiSeq platform, and the raw sequencing data were treated using the QIIME 2 (version 2020.2) pipeline (Bolyen et al., 2019). Operational taxonomic units (OTUs) were generated from raw reads by quality trimming, DADA2 denoising and clustering, and their taxonomy was classified with SILVA database (Quast et al., 2013) by the machine learning software plugin scikit-learn (Pedregosa et al., 2011).

The DNA sequences that were taxonomically characterized within the SAR202 clade were extracted for subsequent subgroup assignment. SAR202 bacteria could be classified into several groups and subgroups based on the genomic sequences (Saw et al., 2020). The 16S rRNA gene sequences were picked from ~500 SAR202 genomes, which were distributed in all known SAR202 groups/subgroups genomes. These selected 16S rRNA sequences were used to build a well-classified SAR202 database which is accessible at this link: https://figshare.com/s/73fe421f9500073e3852. The partial 16S rRNA gene sequences obtained in this study were then blasted against the SAR202 database mentioned above.

### Organic chemical analysis

DOM composition of each subsample was analyzed by FT-ICR-MS described in Zhao et al. (2019). Briefly, DOM from 400 mL GF/F (Whatman®) filtered incubation water was extracted by the solid-phase extraction method (Dittmar et al., 2008). The SPE–DOM samples from the incubations were characterized by a non-targeted ultrahigh-resolution MS approach. All SPE-DOM samples were analyzed at the Helmholtz Center for Environmental Health, Munich, Germany using a 12 Tesla Bruker Solarix Fourier transform ion cyclotron resonance mass spectrometer (FT-ICR MS) interfaced with negative mode ESI. Samples were directly infused into the ionization source at 2 μL/min and injection tubing was rinsed with ~600 μL of a 50% water/methanol mixture after each sample. Formula assignments used in this study are based on the following number of atoms of C_0–70_, O_0–25_, N_0–10_, and S_0–3_, as well as their isotopolgues. After cross-validation, all formulas presented in this study were unambiguous. All MFs that did not exceed a relative intensity threshold of 10^7^ total ion counts (TIC) throughout the incubation were omitted prior to further analysis.

### Network analysis

For both control and treatment samples, two phases of the incubation were determined based on cell abundance and water chemistry profiles (reported in Jia et al., 2023), where day 0 to 30 was phase 1 and day 30 to 364 was phase 2. The 30-day cutoff for these two phases was considered because SAR202 bacteria became relatively more abundant after this point. Moreover, most DOM became semi-labile or semi-recalcitrant after day 30 of incubation (Zheng et al., 2022). To show specific correlations within these two phases, network analysis was performed. Spearman’s rank correlation coefficients between the relative abundance of order-level OTUs and *m/z* ions in both the control and the treatment samples were calculated using the *Hmisc* package of R (version 4.2.0). Significant positive or negative correlations (r ≥ 0.9, *p* < 0.05) were selected and visualized in the network with Cytoscape (version 3.9.1), respectively. The r-value and *p*-value were calculated through the “rcorr” function of the Hmisc package in R. The p-value was calculated based on Student’s t-distribution and multiple-test correction was performed with the Benjamini-Hochberg method using the “p.adjust” function (R codes available at https://github.com/jiayfreddy131/SAR202_network.git).

It needs to be noted that we only used five pairs of subsamples (OTUs and MFs pair) to perform Spearman’s correlation analysis for two separate phases, which would increase the possibility of false correlations. However, we justified the need to separately perform correlation analyses for two phases since we observed large shifts in the microbial community composition. In addition, semi-labile and recalcitrant DOM usually occur in phase 2. Moreover, due to the volume needed for analyses of each subsample (~700 mL), our incubation size was not large enough for more subsamples.

## Results and discussion

### Enrichment of SAR202 in the late incubation phase

**Figure 1:**
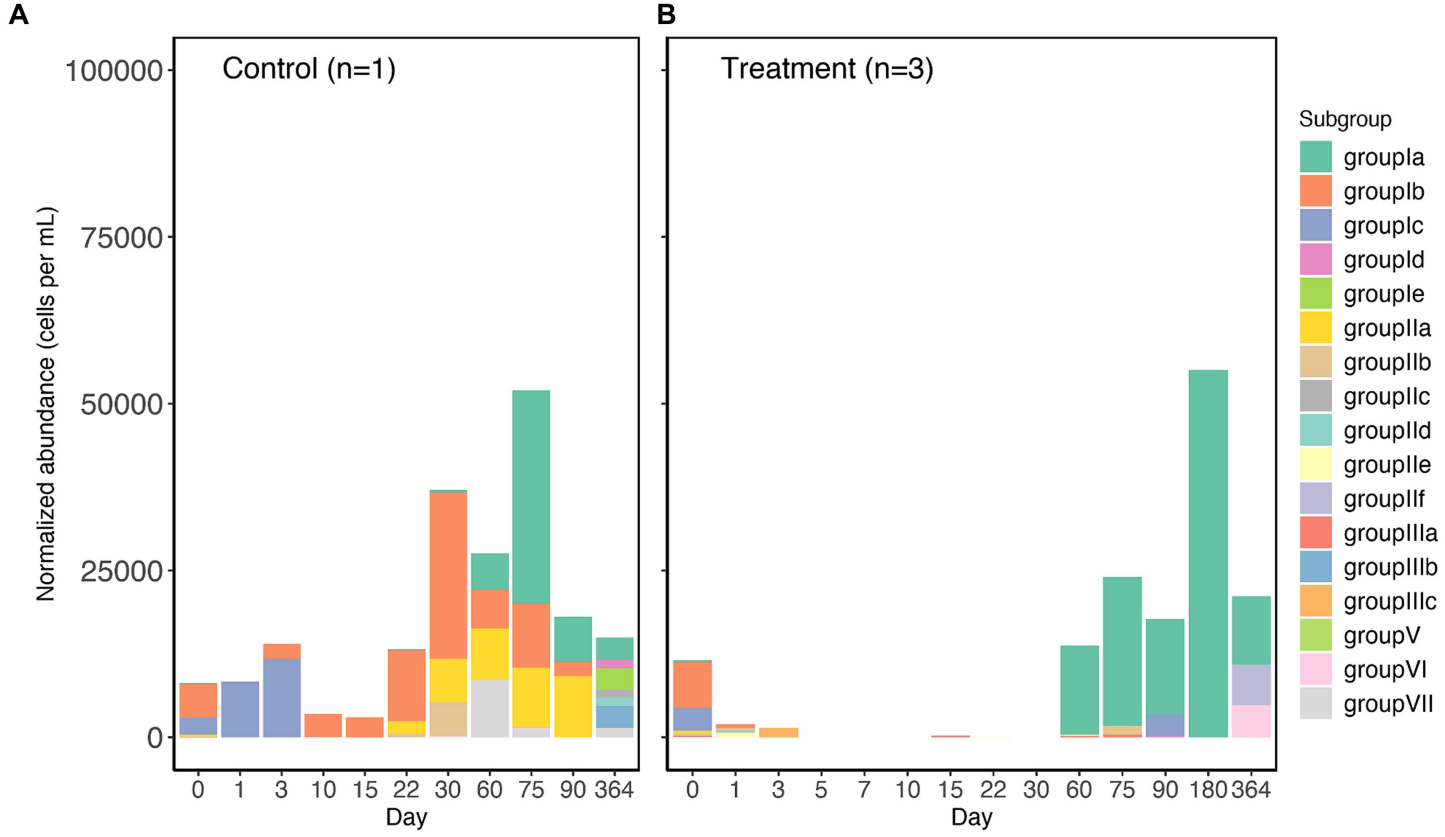
The abundance of subgroups of SAR202 bacteria at different sampling time points (day 0 to 364) of the control **(A)** and treatment **(B)** incubation. Abundance values are normalized to cell abundance recorded by flow cytometry. Treatment values are averages of triplicates. For the control, 3 subsamples (day 5, 7, and 181) were not able to generate positive PCR due to the low microbial biomass of oceanic water.

In the controls and treatment samples, the SAR202 clade on day 0 only comprised less than 12,500 cells per mL (Figures 1A,B), indicating the low relative abundance within the original *in situ* surface seawater. Throughout the one-year incubation, SAR202 in both the controls and the treatments increased in abundance and exceeded 50,000 cells per ml at day 75 and day 180, respectively (Figures 1A,B), likely due to their ability to utilize more resistant DOM molecules (Landry et al., 2017; Mehrshad et al., 2018; Wei et al., 2020). Unfortunately, we were not able to amplify the 16S rRNA gene from many samples in the control group due to the low abundance of microbial cells in the oligotrophic water. The successful samples in the control group are only enough for one replicate with a few sampling points not available. In contrast to the diverse SAR202 subgroups present in the control, SAR202 bacteria in all treatment triplicates were mainly made up of subgroup 1a after day 60 (Figure 1B). This differentiation of subgroup constituents is likely related to the amendment of Syn-DOM in the treatment. This result should be considered preliminary due to the lack of sufficient replicates in the control. Syn-DOM has a “ripple” effect on the microbial composition in the later incubation period, where the initial amendment subsequently influenced the microbial succession, suggesting a cascade of internal cycling of different components of the RDOM pool. This is not surprising, as a similar incubation experiment has shown that the initial addition of *Synechococcus*-derived DOM had a long-term (90 days) effect on the complexity and diversity of the DOM pool (Zhao et al., 2019).

Because we collected our initial microbial community from the surface Atlantic Ocean, the most highly-represented subgroups of SAR202 in our incubations were known to be surface-associated. SAR202 subgroups have diverse enzyme families that contribute to their metabolic specialization (Saw et al., 2020), but the previous SAR202 studies have mainly focused on the metabolic potentials of deep-sea subgroups (Landry et al., 2017; Mehrshad et al., 2018). Meanwhile, the roles that surface subgroups of SAR202 play in the biogeochemical cycle are less known. It has been reported in the pangenomic study that the surface subgroups contain rhodopsin genes in their genomes, and that subgroup I specializes in utilizing chirally complex DOM (Saw et al., 2020). Nonetheless, the powerful oxidative enzymes (e.g., FMNOs) that are encoded by deep-sea lineages of the SAR202 clade are also present in the surface subgroups in fewer copies (Landry et al., 2017; Saw et al., 2020). Recently, several closely related strains of SAR202 bacteria in subgroup Ia were successfully cultivated (Lim et al., 2023). Subsequent growth experiments showed that the subgroup Ia SAR202 did not reach the exponential phase until 50 days at 20°C and the growth responses to fucose and rhamnose were slow and did not reach the highest cell density until the late exponential phase, which coincided with our detection of increased SAR202 abundance in the treatment on day 60 (Figure 1B).

### Strong correspondence between SAR202 and RDOM in the later incubation phase

**Figure 2:**
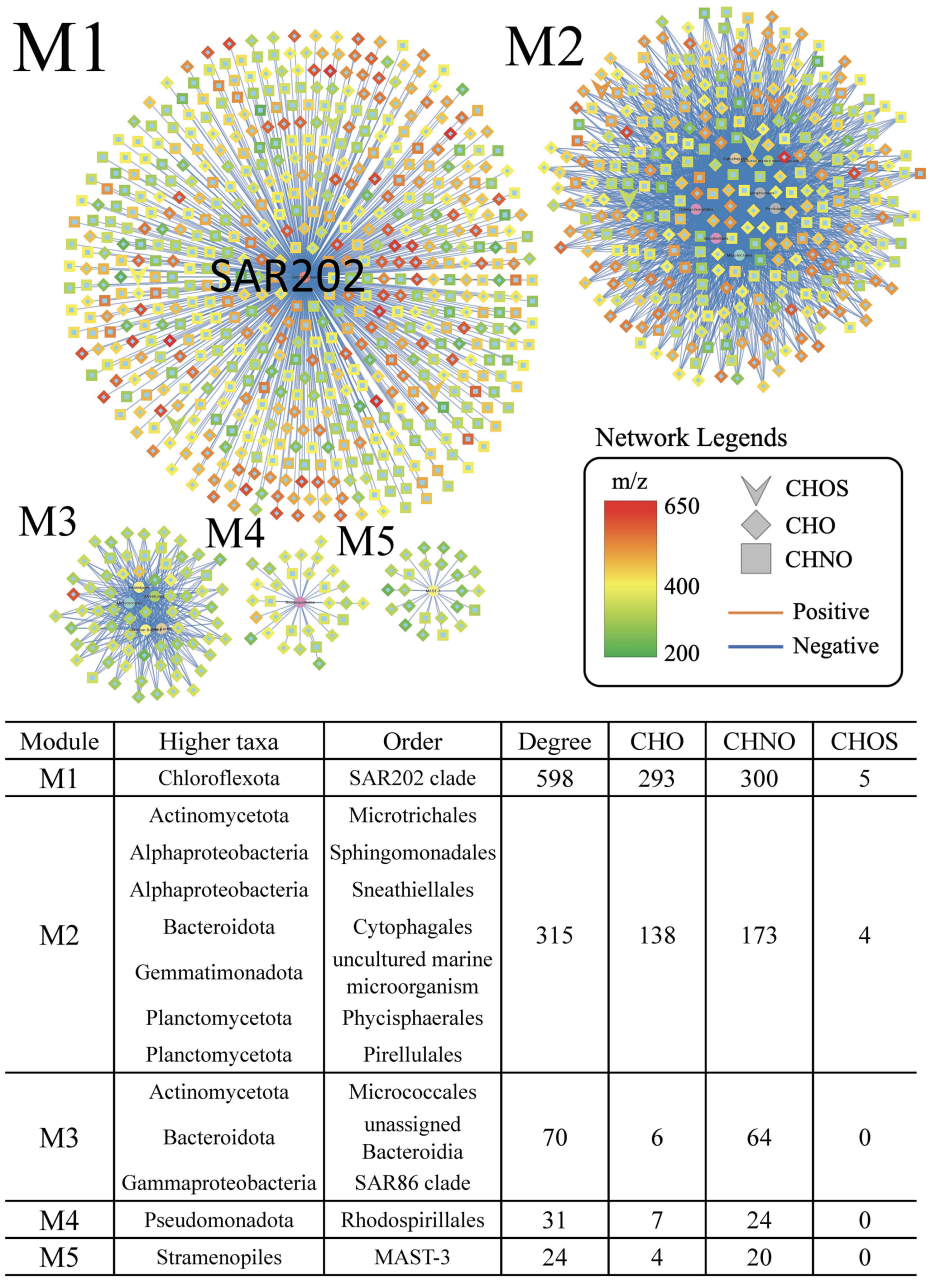
Extracted individual networks showing the statistically significant negative correlations between abundant order-level taxa and DOM formulae in the treatment incubations during phase 2 (day 30–364). The central nodes for various taxa are highlighted. Positive correlations are indicated by red lines, and negative correlations are indicated by blue lines. The nodes represent assigned molecular formulae of CHO (diamond), CHNO (square), and CHOS (arrow). The color gradient of nodes represents the mass-to-charge ratio (m/z) of the formulae from 200 (green) to 400 (yellow) to 650 (red).

To investigate the specific roles of the increasing SAR202 bacteria in the later stage of the incubation, we divided the year-long incubation into 2 phases and we generated network analyses on coordination between bacterial populations and DOM species for both phases separately. The SAR202 clade had the highest degree of correlation in the negative networks of phase 2 (M1 in Figure 2). Other bacterial groups, such as Sphingomonadales, Cytophagales, Micrococcales, and SAR86 were also prevalent in the treatment but they did not have as many associations with MFs as the SAR202 clade (M2–M5 in Figure 2). It is noteworthy that the SAR202 bacteria in the control formed very few associations (n = 6) with MFs (Supplementary Figure S1), suggesting that SAR202 bacteria in the treatment had a strong response to the amended Syn-DOM. SAR202 bacteria did not thrive in phase 1 after the initial amendment of Syn-DOM (Figure 1B), suggesting that they may utilize substrates different from other bacteria in phase 1. After labile DOM was quickly consumed as indicated by the increase of ammonium over time, more semi-labile and recalcitrant DOM likely became available in phase 2. The abundance of SAR202 bacteria increased in phase 2, suggesting that they were able to utilize more resistant DOM. This result supports the specialization of SAR202 in RDOM utilization based on the metagenomic analysis (Landry et al., 2017; Mehrshad et al., 2018; Wei et al., 2020).

### SAR202-associated MFs are more oxygenated and unsaturated

**Figure 3:**
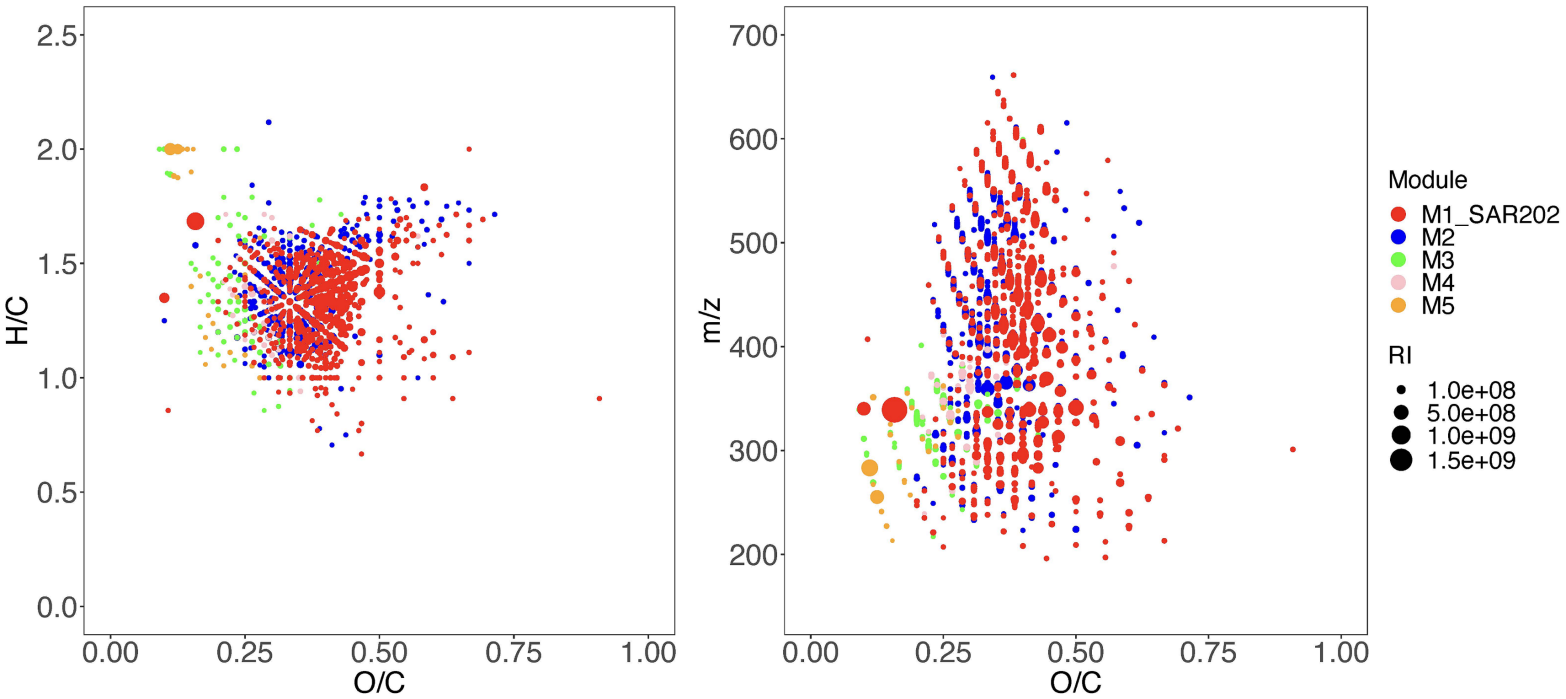
Chemical characteristics of molecular formulae within the top 5 modules in the networks as shown in Figure 2. Both H/C ratio **(A)** and molecular mass (m/z) **(B)** are plotted against the O/C ratio. The molecular formulae from distinct network modules are shown in different colors and the relative intensity values of the molecular formulae at day 30 are indicated by the bubble size.

Given that the SAR202 fell within a large single-taxon network (Figure 1), we wanted to test the uniqueness of the SAR202-associated MFs (SAR202-MFs). We selected the top 5 network modules (M1–M5) based on the degree of associations between the taxa and the MFs (Figure 2) and examined their molecular properties based on the assigned formulae. Compared to MFs in the other modules, SAR202-MFs are positioned at higher O/C ratios and lower H/C ratios, indicating that they are more oxygenated and unsaturated, and they have on average higher molecular weights (*t-*test, *p* < 0.05; Figures 3A,B). Moreover, the abundance of SAR202-MFs is generally higher than that in the other modules, indicated by the bubble sizes of individual MFs (Figure 3). This suggests that SAR202 bacteria are specifically utilizing a unique niche of high-abundance unsaturated compounds that were either not consumed by other bacteria or produced by bacteria in phase 1. It has been postulated that SAR202 bacteria are capable of exploiting the DOM that other deep-sea bacterioplankton cannot degrade (Varela et al., 2008; Lim et al., 2023). Through the ultrahigh-resolution FT-ICR-MS approach, our data provided first-hand evidence that the SAR202-MFs possess possibly aromatic but certainly unsaturated characteristics.

### SAR202-MFs were produced in phase 1

**Figure 4:**
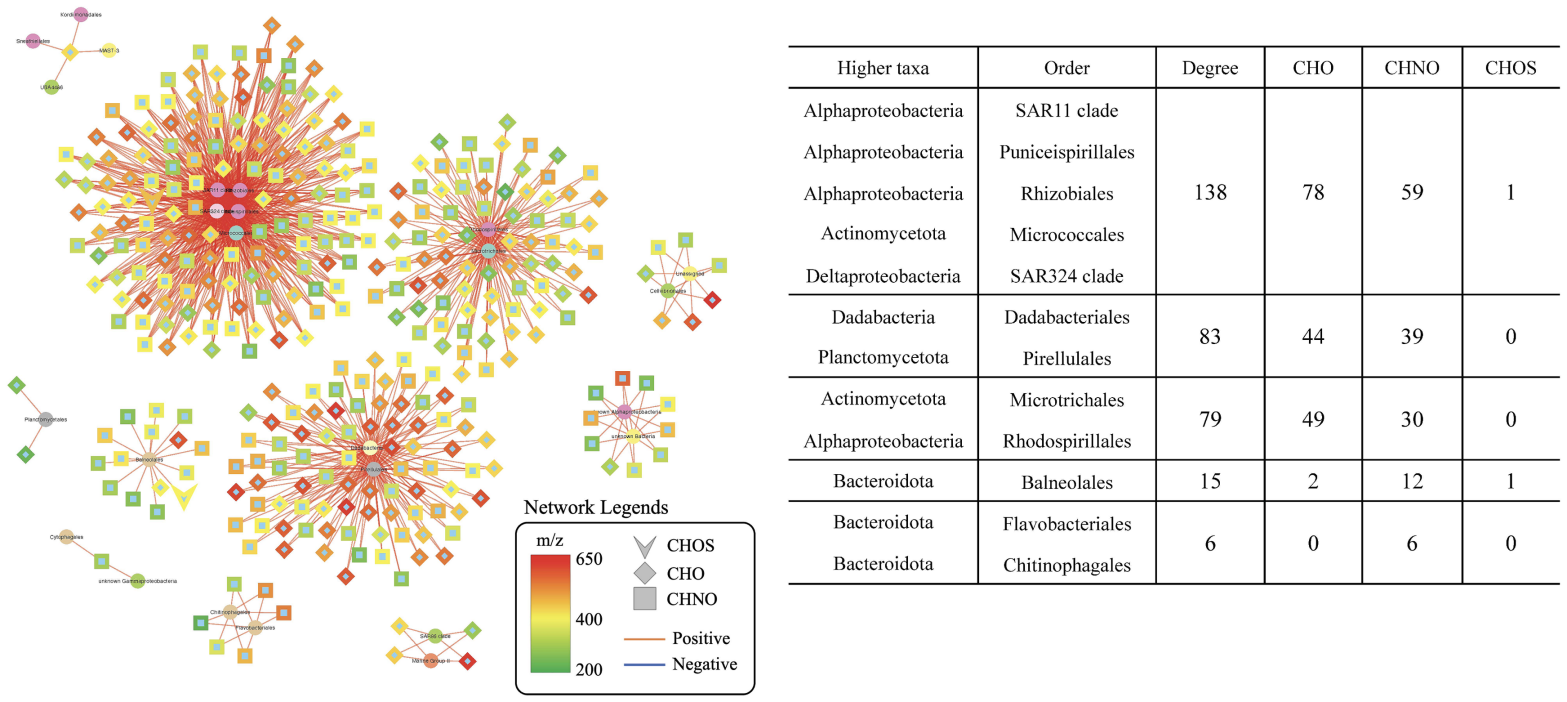
Extracted networks showing the statistically significant positive correlations between order-level taxa and SAR202-associated molecular formulae in the treatment incubation during phase 1 (day 0–30). Network legends are identical to that of Figure 2.

The finding of significant correlations between SAR202 and specific MFs in phase 2 led to a hypothesize that these SAR202-associated MFs were produced in phase 1 via the heterotrophic activity. We searched for the relationship between SAR202-associated MFs (identified in phase 2) and bacterial populations in phase 1 using network analysis. Out of 598 SAR202-associated MFs (identified in phase 2), 346 MFs have at least one positive correlation with one bacterial taxa (Figure 4), suggesting that about 60% of SAR202-associated MFs are potentially produced by heterotrophic activities in phase 1. Within the phase 1 positive network, the SAR202-MFs accounted for 11.2% (346 out of 3,089), and were correlated with 9.0% of total taxa (13 out of 145). Previous incubation experiments have concluded that bacteria can produce “recalcitrant” DOM in the later phase of incubation (Zhao et al., 2019; Zheng et al., 2021). Here, we demonstrated that the production of semi-labile and recalcitrant DOM is associated with different groups of heterotrophic bacteria in the early stage of incubation. We demonstrated that different groups of the bacterial community work together to utilize and transform Syn-DOM during the incubation period. DOM produced in phase 1 (<30 days) may not be as recalcitrant as previously assumed and certain specialists such as SAR202 bacteria can continue utilizing this intermediate DOM. Although increasing evidence points to the potential role of SAR202 in the ocean’s RDOM cycling, much more research is needed to further understand actual activities and rates of processes of SAR202 in the ocean.

**Figure 5:**
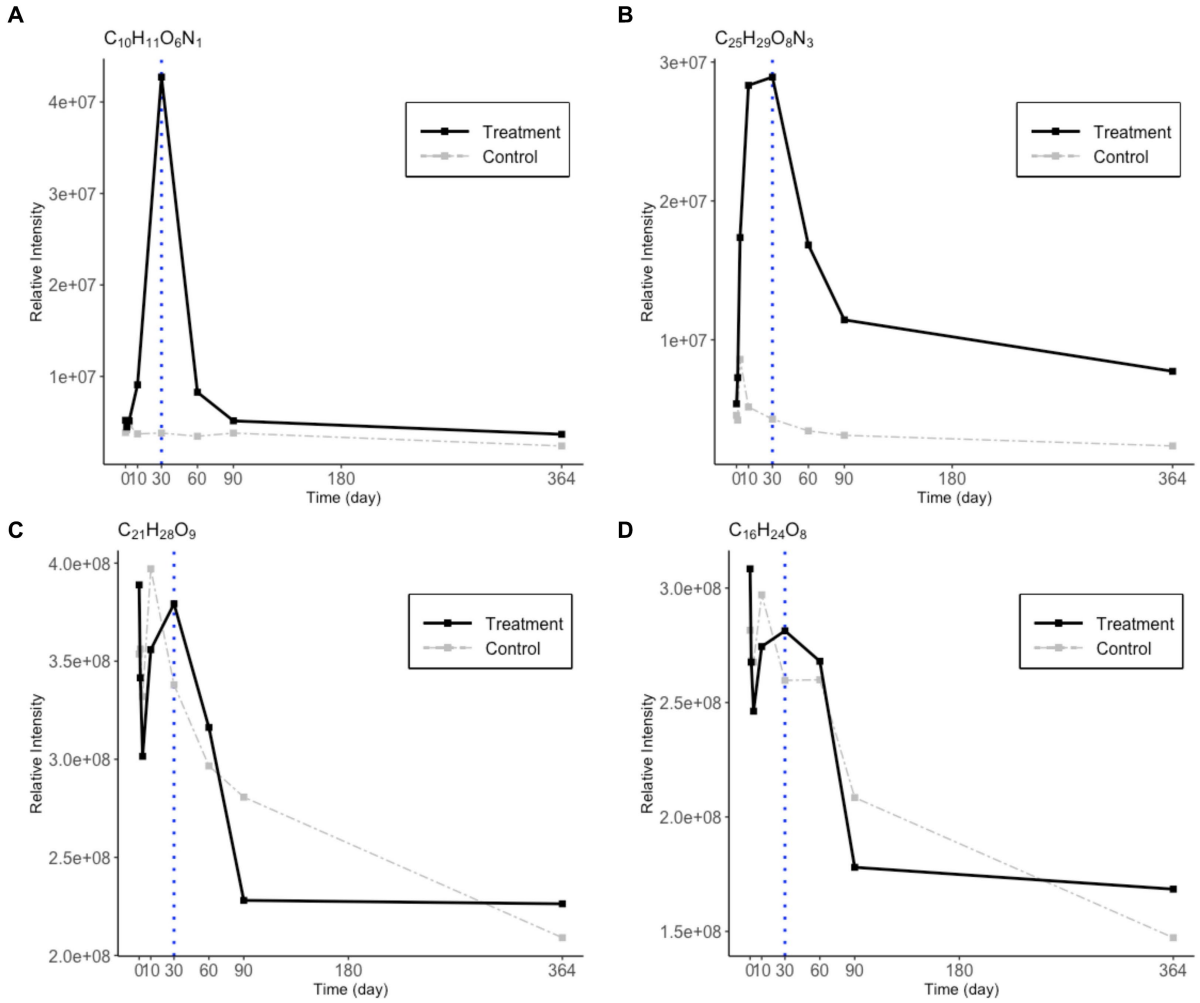
Changes in relative intensity values for 2 CHNO **(A,B)** and 2 CHO **(C,D)** representative molecular formulae during the one-year incubation. The treatment values are in black solid lines and the control values are in gray dashed lines. A blue dotted line is drawn on day 30 to indicate the break point between phase 1 and 2.

It is noteworthy that microbial degradation of N-rich DOM is different from that of N-free DOM during the incubation (Figure 5). The two representative CHON compounds were present in low abundance at the beginning, increased from day 0 to day 30, and decreased after day 30. The two representative CHO compounds started with high concentrations and exhibited different degrading patterns than the CHON compounds. This indicates that the CHNO and CHO compounds that SAR202 utilized in phase 2 originated from varied sources. The CHNO formulae were accumulated following the bacterial utilization of the Syn-DOM materials, while the CHO formulae were more likely from the *in situ* seawater.

Metagenomic studies have suggested a potential role of SAR202 in RDOC cycling (Landry et al., 2017), previous experiments have focused on providing direct evidence of the SAR202 utilization of less labile DOC. SAR202 has been reported to have a significant response to the amendment of lignin, which is a recalcitrant mixture of polyphenols (Liu et al., 2020b). This characteristic may be related to their connections with CHO MFs in the network (Figures 5C,D). However, the N-containing DOM has not been paid much attention as previous incubation studies determined that the DON pool is quickly consumed and is rather labile (Zhao et al., 2019; Zheng et al., 2021). A recent study using stable isotope probes identified SAR202’s ability to incorporate DOM derived from *Synechococcus* lysate (Liu et al., 2020a), which has been shown to contain a complex and diverse group of N-rich molecules (Zhao et al., 2019; Zheng et al., 2021). According to our data, a portion of N-containing DOM accumulated during phase 1 before they decreased in abundance in phase 2 (Figures 5A,B). These “intermediate” DON molecules may be part of the production of the heterotrophic activity on the initial *Synechococcus* lysate amendment in phase 1 (Figure 4). Moreover, this pool of specific DON cannot be quickly utilized by other bacteria, which suggests their refractory characteristic in phase 1. DOM within such time-series trends has been ignored in previous incubation studies, as the complex DOM pool was examined as a whole rather than individually. The discovery of these SAR202-correlated CHNO MFs is intriguing since they represent a group of “intermediate DOM” within the incubation system that could drive a second biogeochemical cycle in the later phase, which was overlooked by previous incubation studies. It also hints toward a pool of DON that is largely resistant to heterotrophic bacteria that rely on labile DOM but that seems to be an energy source for SAR202. A substantial pool of DON molecular signatures has always been reported in high-resolution MS in the deep ocean but its sources remained unknown. Our experiments support the previous genomic predictions that SAR202 possesses the enzymatic ability to take up recalcitrant DOM, and we further elucidated that the correlation between SAR202 bacteria and refractory DOM over time using a microcosm incubation experiment.

## Data availability statement

The datasets presented in this study can be found in online repositories. The names of the repository/repositories and accession number(s) can be found in the article/Supplementary material.

## Author contributions

YJ: Data curation, Formal analysis, Investigation, Methodology, Software, Writing – original draft, Visualization. CH: Formal analysis, Methodology, Software, Writing – review & editing. ML: Formal analysis, Methodology, Writing – review & editing. QC: Methodology, Writing – review & editing. LP: Methodology, Writing – review & editing. MG: Conceptualization, Data curation, Formal analysis, Funding acquisition, Methodology, Project administration, Resources, Supervision, Validation, Writing – review & editing. FC: Conceptualization, Funding acquisition, Project administration, Resources, Supervision, Validation, Writing – review & editing.

## References

[ref1] Apprill A. Mcnally S. Parsons R. Weber L. (2015). Minor revision to V4 region SSU rRNA 806R gene primer greatly increases detection of SAR11 bacterioplankton. Aquat. Microb. Ecol. 75, 129–137. doi: 10.3354/ame01753

[ref2] Azam F. Fenchel T. Field J. G. Gray J. S. Meyer-Reil L. A. Thingstad F. (1983). The ecological role of water-column microbes in the sea. Mar. Ecol. Prog. Ser. 10, 257–263. doi: 10.3354/meps010257

[ref3] Batushansky A. Toubiana D. Fait A. (2016). Correlation-based network generation, visualization, and analysis as a powerful tool in biological studies: a case study in Cancer cell metabolism. Biomed. Res. Int. 2016:8313272. doi: 10.1155/2016/8313272 PMID: 27840831 PMC5090126

[ref4] Bolyen E. Rideout J. R. Dillon M. R. Bokulich N. A. Abnet C. C. Al-Ghalith G. A.. (2019). Reproducible, interactive, scalable and extensible microbiome data science using QIIME 2. Nat. Biotechnol. 37, 852–857. doi: 10.1038/s41587-019-0209-9, PMID: 31341288 PMC7015180

[ref5] Cardona C. Weisenhorn P. Henry C. Gilbert J. A. (2016). Network-based metabolic analysis and microbial community modeling. Curr. Opin. Microbiol. 31, 124–131. doi: 10.1016/j.mib.2016.03.008, PMID: 27060776

[ref6] Colatriano D. Tran P. Q. Guéguen C. Williams W. J. Lovejoy C. Walsh D. A. (2018). Genomic evidence for the degradation of terrestrial organic matter by pelagic Arctic Ocean Chloroflexi bacteria. Commun. Biol. 1:90. doi: 10.1038/s42003-018-0086-7, PMID: 30271971 PMC6123686

[ref7] DeLong E. F. Preston C. M. Mincer T. Rich V. Hallam S. J. Frigaard N.-U.. (2006). Community genomics among stratified microbial assemblages in the ocean’s interior. Science 311, 496–503. doi: 10.1126/science.1120250, PMID: 16439655

[ref8] Dittmar T. (2015). Reasons behind the long-term stability of dissolved organic matter. In: Hansell D. A. Carlson C. A., (eds). Biogeochemistry of marine dissolved organic matter. (San Diego, CA: Academic Press); 2015. pp. 369–385.

[ref9] Dittmar T. Koch B. Hertkorn N. (2008). A simple and efficient method for the solid-phase extraction of dissolved organic matter (SPE-DOM) from seawater. Limnol. Oceanogr. Methods 6, 230–235. doi: 10.4319/lom.2008.6.230

[ref10] Gao Z. M. Huang J. M. Cui G. J. Li W. L. Li J. Wei Z. F.. (2019). In situ meta-omic insights into the community compositions and ecological roles of hadal microbes in the Mariana trench. Environ. Microbiol. 21, 4092–4108. doi: 10.1111/1462-2920.14759, PMID: 31344308

[ref11] Giovannoni S. J. Rappei M. S. Vergin K. L. Adair N. L. (1996). 16S rRNA genes reveal stratified open ocean bacterioplankton populations related to the green non-sulfur bacteria. Proc. Natl. Acad. Sci. U. S. A. 93, 7979–7984. doi: 10.1073/pnas.93.15.7979, PMID: 8755588 PMC38860

[ref12] Hansell D. A. (2013). Recalcitrant dissolved organic carbon fractions. Ann. Rev. Mar. Sci. 5, 421–445. doi: 10.1146/annurev-marine-120710-100757 22881353

[ref13] Hansell D. A. Carlson C. A. Repeta D. J. Reiner S. (2009). Dissolved organic matter in the ocean. Oceanography 22, 202–211. doi: 10.5670/oceanog.2009.109

[ref14] Jia Y. Lahm M. Chen Q. Powers L. Gonsior M. Chen F. (2023). The predominance of Ammonia-oxidizing Archaea in an oceanic microbial community amended with cyanobacterial lysate. Microbiol. Spectr. 11, e02405–e02422. doi: 10.1128/spectrum.02405-22 36622233 PMC9927567

[ref15] Jiao N. Cai R. Zheng Q. Tang K. Liu J. Jiao F.. (2018). Unveiling the enigma of refractory carbon in the ocean. Natl. Sci. Rev. 5, 459–463. doi: 10.1093/nsr/nwy020

[ref16] Jiao N. Herndl G. J. Hansell D. A. Benner R. Kattner G. Wilhelm S. W.. (2010). Microbial production of recalcitrant dissolved organic matter: long-term carbon storage in the global ocean. Nat. Rev. Microbiol. 8, 593–599. doi: 10.1038/nrmicro2386, PMID: 20601964

[ref17] Kan J. (2006). Bacterioplankton in the Chesapeake Bay: Genetic diversity, population dynamics, and community proteomics. Doctoral dissertation. University of Maryland, College Park, MD, USA.

[ref18] Kan J. Wang K. Chen F. (2006). Temporal variation and detection limit of an estuarine bacterioplankton community analyzed by denaturing gradient gel electrophoresis (DGGE). Aquat. Microb. Ecol. 42, 7–18. doi: 10.3354/ame042007

[ref19] Kirchman D. L. Lancelot C. Fasham M. Legendre L. Radach G. Scott M. (1993). “Dissolved organic matter in biogeochemical models of the ocean” in Evans G. T. Fasham M. J. R., (eds). Towards a model of ocean biogeochemical processes (Berlin: Springer-Verlag), 209–225.

[ref20] Landry Z. Swan B. K. Herndl G. J. Stepanauskas R. Giovannoni S. J. (2017). SAR202 genomes from the dark ocean predict pathways for the oxidation of recalcitrant dissolved organic matter. MBio 8, e00413–e00417. doi: 10.1128/mBio.00413-17 28420738 PMC5395668

[ref21] Lim Y. Seo J. H. Giovannoni S. J. Kang I. Cho J. C. (2023). Cultivation of marine bacteria of the SAR202 clade. Nat. Commun. 14:5098. doi: 10.1038/s41467-023-40726-8, PMID: 37607927 PMC10444878

[ref22] Liu S. Baetge N. Comstock J. Opalk K. Parsons R. Halewood E.. (2020a). Stable isotope probing identifies Bacterioplankton lineages capable of utilizing dissolved organic matter across a range of bioavailability. Front. Microbiol. 11, 1–27. doi: 10.3389/fmicb.2020.580397 33117322 PMC7575717

[ref23] Liu S. Parsons R. Opalk K. Baetge N. Giovannoni S. Bolaños L. M.. (2020b). Different carboxyl-rich alicyclic molecules proxy compounds select distinct bacterioplankton for oxidation of dissolved organic matter in the mesopelagic Sargasso Sea. Limnol. Oceanogr. 65, 1532–1553. doi: 10.1002/lno.11405

[ref24] Luria C. M. Amaral-Zettler L. A. Ducklow H. W. Repeta D. J. Rhyne A. L. Rich J. J. (2017). Seasonal shifts in bacterial community responses to phytoplankton-derived dissolved organic matter in the Western Antarctic peninsula. Front. Microbiol. 8:2117. doi: 10.3389/fmicb.2017.02117, PMID: 29163409 PMC5675858

[ref25] Mehrshad M. Rodriguez-Valera F. Amoozegar M. A. López-García P. Ghai R. (2018). The enigmatic SAR202 cluster up close: shedding light on a globally distributed dark ocean lineage involved in sulfur cycling. ISME J. 12, 655–668. doi: 10.1038/s41396-017-0009-5, PMID: 29208946 PMC5864207

[ref26] Moran M. A. Kujawinski E. B. Stubbins A. Fatland R. Aluwihare L. I. Buchan A.. (2016). Deciphering Ocean carbon in a changing world. Proc. Natl. Acad. Sci. U. S. A. 113, 3143–3151. doi: 10.1073/pnas.1514645113, PMID: 26951682 PMC4812754

[ref27] Morris R. M. Rappé M. S. Urbach E. Connon S. A. Giovannoni S. J. (2004). Prevalence of the Chloroflexi-related SAR202 bacterioplankton cluster throughout the mesopelagic zone and deep ocean. Appl. Environ. Microbiol. 70, 2836–2842. doi: 10.1128/AEM.70.5.2836-2842.2004, PMID: 15128540 PMC404461

[ref28] Parada A. E. Needham D. M. Fuhrman J. A. (2016). Every base matters: assessing small subunit rRNA primers for marine microbiomes with mock communities, time series and global field samples. Environ. Microbiol. 18, 1403–1414. doi: 10.1111/1462-2920.13023, PMID: 26271760

[ref29] Pedregosa F. Varoquaux G. Gramfort A. Michel V. Thirion B. Grisel O.. (2011). Scikit-learn: machine learning in Python. J. Mach. Learn. Res. 12, 2825–2830. doi: 10.48550/arXiv.1201.0490

[ref30] Quast C. Pruesse E. Yilmaz P. Gerken J. Schweer T. Yarza P.. (2013). The SILVA ribosomal RNA gene database project: improved data processing and web-based tools. Nucleic Acids Res. 41, D590–D596. doi: 10.1093/nar/gks1219 23193283 PMC3531112

[ref31] Raina J. B. Lambert B. S. Parks D. H. Rinke C. Siboni N. Bramucci A.. (2022). Chemotaxis shapes the microscale organization of the ocean’s microbiome. Nature 605, 132–138. doi: 10.1038/s41586-022-04614-3, PMID: 35444277

[ref32] Saw J. H. W. Nunoura T. Hirai M. Takaki Y. Parsons R. Michelsen M.. (2020). Pangenomics analysis reveals diversification of enzyme families and niche specialization in globally abundant SAR202 bacteria. MBio 11, e02975–e02919. doi: 10.1128/mBio.02975-19 31911493 PMC6946804

[ref33] Schattenhofer M. Fuchs B. M. Amann R. Zubkov M. Tarran G. A. Pernthaler J. (2009). Latitudinal distribution of prokaryotic picoplankton populations in the Atlantic Ocean. Environ. Microbiol. 11, 2078–2093. doi: 10.1111/j.1462-2920.2009.01929.x, PMID: 19453607

[ref34] Varela M. M. van Aken H. M. Herndl G. J. (2008). Abundance and activity of Chloroflexi-type SAR202 bacterioplankton in the meso- and bathypelagic waters of the (sub)tropical Atlantic. Environ. Microbiol. 10, 1903–1911. doi: 10.1111/j.1462-2920.2008.01627.x, PMID: 18422640

[ref35] Wang Y. Xie R. Shen Y. Cai R. He C. Chen Q.. (2022). Linking microbial population succession and DOM molecular changes in Synechococcus-derived organic matter addition incubation. Microbiol. Spectr. 10, e02308–e02321. doi: 10.1128/spectrum.02308-21 35380472 PMC9045170

[ref36] Wei Z. F. Li W. L. Huang J. M. Wang Y. (2020). Metagenomic studies of SAR202 bacteria at the full-ocean depth in the Mariana trench. Deep-Sea Res. I Oceanogr. Res. Pap. 165:103396. doi: 10.1016/j.dsr.2020.103396

[ref37] Xiao X. Guo W. Li X. Wang C. Chen X. Lin X.. (2021). Viral lysis alters the optical properties and biological availability of dissolved organic matter derived from Prochlorococcus picocyanobacteria. Appl. Environ. Microbiol. 87, e02271–e02220. doi: 10.1128/AEM.02271-20 33218998 PMC7848921

[ref38] Xie R. Wang Y. Chen Q. Guo W. Jiao N. Zheng Q. (2020). Coupling between carbon and nitrogen metabolic processes mediated by coastal microbes in *Synechococcus*-derived. Front. Microbiol. 11:1041. doi: 10.3389/fmicb.2020.01041 32523578 PMC7261836

[ref39] Zhang C. Dang H. Azam F. Benner R. Legendre L. Passow U.. (2018). Evolving paradigms in biological carbon cycling in the ocean. Natl. Sci. Rev. 5, 481–499. doi: 10.1093/nsr/nwy074

[ref40] Zhao Z. Gonsior M. Schmitt-Kopplin P. Zhan Y. Zhang R. Jiao N.. (2019). Microbial transformation of virus-induced dissolved organic matter from picocyanobacteria: coupling of bacterial diversity and DOM chemodiversity. ISME J. 13, 2551–2565. doi: 10.1038/s41396-019-0449-1, PMID: 31227815 PMC6776026

[ref41] Zheng X. X. Cai R. Yao H. W. Zhuo X. C. He C. Zheng Q.. (2022). Experimental insight into the enigmatic persistence of marine refractory dissolved organic matter. Environ. Sci. Technol. 56, 17420–17429. doi: 10.1021/acs.est.2c04136, PMID: 36347804

[ref42] Zheng Q. Chen Q. Cai R. He C. Guo W. Wang Y.. (2019). Molecular characteristics of microbially mediated transformations of Synechococcus-derived dissolved organic matter as revealed by incubation experiments. Environ. Microbiol. 21, 2533–2543. doi: 10.1111/1462-2920.14646, PMID: 31044472

[ref43] Zheng Q. Lin W. Wang Y. Li Y. He C. Shen Y.. (2021). Highly enriched N-containing organic molecules of Synechococcus lysates and their rapid transformation by heterotrophic bacteria. Limnol. Oceanogr. 66, 335–348. doi: 10.1002/lno.11608

[ref44] Zhou J. Richlen M. L. Sehein T. R. Kulis D. M. Anderson D. M. Cai Z. (2018). Microbial community structure and associations during a marine dinoflagellate bloom. Front. Microbiol. 9:1201. doi: 10.3389/fmicb.2018.01201, PMID: 29928265 PMC5998739

[ref45] Zhou L. Zhou Y. Tang X. Zhang Y. Jang K. S. Székely A. J.. (2021). Resource aromaticity affects bacterial community successions in response to different sources of dissolved organic matter. Water Res. 190:116776. doi: 10.1016/j.watres.2020.116776, PMID: 33387955

