# Supplementary material for: A pilot study suggests the correspondence between SAR202 bacteria and dissolved organic matter in the late stage of a year-long microcosm incubation
Source: Front Microbiol. 2024 Apr 3;15:1357822. doi: 10.3389/fmicb.2024.1357822 (PMC11021592; doi:10.3389/fmicb.2024.1357822)
Supplement: Supplementary file 2 [file Data_Sheet_1.DOCX]

Supporting Information

**Strong correspondence between SAR202 bacteria and dissolved organic matter occurs in the late stage of a year-long microcosm incubation**

**Figure S1** Extracted individual networks showing the statistically significant correlations between abundant order-level taxa and DOM formulae in the control incubations during phase 2 (day 30-364). The central nodes for various taxa are highlighted. Positive correlations are indicated by red lines, and negative correlations are indicated by blue lines. The nodes represent assigned molecular formulae of CHO (diamond), CHNO (square), and CHOS (arrow). The color gradient of nodes represents the mass-to-charge ratio (m/z) of the formulae from 200 (green) to 400 (yellow) to 650 (red).

**Table S1** Molecular information of all SAR202-associated molecular formulae.
